# In Vitro Degradation Behaviors of Manganese-Calcium Phosphate Coatings on an Mg-Ca-Zn Alloy

**DOI:** 10.1155/2018/6268579

**Published:** 2018-02-13

**Authors:** Yichang Su, Yingchao Su, Wei Zai, Guangyu Li, Cuie Wen

**Affiliations:** ^1^Key Laboratory of Automobile Materials, Ministry of Education, College of Materials Science and Engineering, Jilin University, Changchun 130025, China; ^2^School of Engineering, RMIT University, Melbourne, VIC 3001, Australia

## Abstract

In order to decrease the degradation rate of magnesium (Mg) alloys for the potential orthopedic applications, manganese-calcium phosphate coatings were prepared on an Mg-Ca-Zn alloy in calcium phosphating solutions with different addition of Mn^2+^. Influence of Mn content on degradation behaviors of phosphate coatings in the simulated body fluid was investigated to obtain the optimum coating. With the increasing Mn addition, the corrosion resistance of the manganese-calcium phosphate coatings was gradually improved. The optimum coating prepared in solution containing 0.05 mol/L Mn^2+^ had a uniform and compact microstructure and was composed of MnHPO_4_·3H_2_O, CaHPO_4_·2H_2_O, and Ca_3_(PO_4_)_2_. The electrochemical corrosion test in simulated body fluid revealed that polarization resistance of the optimum coating is 36273 Ωcm^2^, which is about 11 times higher than that of phosphate coating without Mn addition. The optimum coating also showed the most stable surface structure and lowest hydrogen release in the immersion test in simulated body fluid.

## 1. Introduction

Orthopedic surgery in recent times depends profoundly on the development of biomaterials used for fixation of fractures and joint replacement [1]. Among the three main kinds of biological implant materials, metallic materials, ceramic materials, and polymeric materials, biodegradable metals and polymers have gained interest for their advantages of being gradually dissolved, absorbed, consumed, or excreted in the human body, so there is no need for the secondary surgery to remove implants after the surgery regions have healed [2]. Current biodegradable implants made of polymers have an unsatisfactory mechanical strength [3] and therefore limited applications. Thus, magnesium (Mg) and its alloys have been attracting growing attention as next-generation medical material suitable for biodegradable bone implant and stent due to their well physical and mechanical properties, such as excellent biocompatibility, high strength, and similar elastic modulus to human bone [4–7]. However, the rapid decomposition speed of Mg alloys hinders the implants to fulfill their surgical function before being discharged, the inhomogeneous local corrosion starting from the surface of Mg alloys makes the corrosion behavior uncontrollable, and too much hydrogen evolved can be accumulated in gas pockets next to the corroding Mg implant, which will delay healing of the surgery region and lead to inflammatory reaction.

Attempts to improve the corrosion resistance performance have been made on various Mg alloys, such as on AZ91 [8, 9], AZ31 [10, 11], AM60 [12], ZM21 [13], Mg-Ca [14], Mg-Zn and Mg-Mn [15], Mg-Zr-Sr [16], and Mg-Zn-Ca. Among these Mg based alloys, AZ91 and AM60 contain Al which is indicated in several pathological conditions in the human, the most commonly associated ones include dementia and Alzheimer's disease [17]. Ca was a preferable addition element for its capability of refining microstructure and biomimetic mineralization behavior during alloy biodegradable process [18]; Zn is next to aluminum in strengthening effectiveness as an alloying element in Mg, and adding Zn can improve both the tensile strength and the corrosion resistance of Mg alloys [19]. In vitro cytotoxicity assessments indicated that Mg-Zn-Ca alloys did not induce toxicity in L-929 cells and are suitable for biomedical applications [20].

Surface treatment considered in previous studies includes chemical conversion coatings [21–23], electrochemical plating [24], anodizing [25], microarc [26], polymer coatings [27], and sol-gel coatings [28]. Among all the treatment methods, phosphate conversion coating was easier to acquire and more environmentally friendly. Phosphate conversion coating consists of crystalline or amorphous surface metal phosphates or of metal phosphate ions in the passivating solution and is usually carried out by immersion of the metal samples into the phosphating baths at a certain range of bath temperature and pH value of the bath solution [29, 30]. Besides, when phosphate coating is used as a pretreatment layer between the substrate and a top layer, the pores in the coating may improve the bonding ability between substrate and top coating [31]. Calcium phosphate (CaP) coatings enhance cellular adhesion, proliferation, and differentiation to promote bone regeneration [22, 32–35]. Studies also referred to the fact that increase of calcium phosphate could maintain homeostasis and reduce the level of pH in physiological system [36]. Manganese-containing coatings induced higher bone-related gene expression in a culture of osteoblastic cells. This indicates that manganese could improve cell mediated mineralization [37]. CaP coating was reported in our previous work and coatings containing Mn element were reported in studies elsewhere that contributed to corrosion resistance on Mg alloys [24, 38–40].

Calcium can significantly influence the stability of Mg-Zn alloy, the crystallization of alloy can be refined [41], and a precipitate that has a complex structure can form. The precipitation has good high temperature stability, which can significantly improve the creep resistance of Mg alloy. The addition of trace amounts of Ca can have a substantial positive influence on the precipitation, process during artificial ageing in the Mg-Zn system [41]. The microstructure of the modified alloy is more complex and has a much refined precipitate structure that is very stable for prolonged periods, leading to a significant improvement in creep resistance. Mg-5Zn-1.5Ca alloy was found to be nontoxic and have good mechanical properties (the comparison is in another unpublished article of our study); thus Mg-5Zn-1.5Ca alloy was applied as Mg matrix substrate in this study.

The aim of this study was to develop a manganese-calcium phosphate conversion coating on Mg-5Zn-1.5Ca alloy which only employed MnCl_2_ and Ca (NO_3_)_2_ as the essential precursors that further improve the corrosion resistance property. Effect of Mn addition's amount in the phosphate solution to the coating's corrosion resistance was investigated; the in vitro biodegradation behavior and hydrogen evolution in SBF were evaluated. The morphology and composition of the coatings were also examined by X-ray diffraction (XRD) and scanning electron microscope (SEM) with energy dispersion spectroscopy (EDS). Furthermore, we also analyzed the polarization measurement, degradation behavior, impedance, and anticorrosion ability of a conversion coating on the Mg alloy.

## 2. Materials and Methods

### 2.1. Sample Preparation and Coating Process

In this work, a home-made extruded Mg-5Zn-1.5Ca alloy with a dimension of 10 mm × 10 mm × 5 mm and compositions of 5 wt% Zn, 1.5 wt% Ca, and Mg balance was used as the substrate material in the following coating processes. Firstly, the samples were subsequently abraded by 500, 1000, 1500, and 2000 grit sandpapers to remove the oxide layer and to obtain similar surface roughness not more than RA 0.05 (the phosphate coatings would be uneven if the surface roughness of the Mg alloy substrate is high) and then ultrasonically rinsed in ethanol in order to remove grease on the alloy surface. For comparison purpose, 3 sets of samples were prepared and tested, respectively, and the average values were calculated. In this study, a “calcium salts pretreatment and phosphate coating” phosphate process was introduced to prepare phosphate coatings. The purpose of calcium salts pretreatment was to form denser and homogenous calcium activated particle on the sample surface which contributes to the uniform and complete phosphate coatings.

Samples were pretreated in a calcium solution with 1.1 mol/L CaO and 0.20 mol/L HNO3 liquor for 0.5 minutes at pH 1.8. Then the samples were rinsed with deionized water and were coated in the phosphate solution for 10 minutes under 37°C at pH 2.7. The composition of phosphate solution was listed in Table 1. The pH of the phosphating solution was adjusted using nitric acid (HNO3) or sodium hydroxide (NaOH). All the chemical reagents were of reagent grade and purchased from Sinopharm Chemical Reagent Co. (Shanghai, China).

### 2.2. Analysis of Composition of Phosphate Coating and Morphology Analysis

The phases of the coatings were analyzed by a X-ray diffractometer (XRD, Rigaku Dymax, Japan) with a Cu K*α* radiation (*γ* = 0.154178 nm) and a monochromator at 40 kV and 100 mA with the scanning rate and step being 4°/min and 0.02°, respectively. The surface morphologies of the samples were examined using a JSM-5310 scanning electron microscope (SEM, ZEISS EV018, Germany), with the voltage of 20 kV and working distance (WD) of 10.0 mm. The surface chemical elements were analyzed using an attached INC250 energy dispersive X-ray analyzer (EDS, X-Max, Oxford Instruments, UK) with the acquisition duration of 60 s.

The surface morphologies of the samples were examined using a JSM-5310 scanning electron microscope (SEM, ZEISS EV018, Germany; WD = 12.0, EHT = 20 kV). After performing immersion test in SBF for 168 hours, the surface morphologies of the samples were characterized and analyzed.

### 2.3. Potentiodynamic Polarization

The electrochemical measurements were performed by using a potentiostat (VersaSTAT 3, Princeton Applied Research, US) with a three-electrode system. In this system, a platinum electrode was treated as the counter electrode, and a saturated calomel electrode (SCE, +0.242 V versus SHE) was regarded as the reference electrode, while the sample with an exposed area of 1 cm^2^ was regarded as the working electrode. Prior to performing the measurements, the samples were immersed in the SBF solution for about 30 min to establish an open circuit potential (OCP) for making the system stable firstly.

### 2.4. Electrochemical Impedance Spectroscopy

The corrosion resistance and biodegradable property of the coatings were evaluated by the electrochemical measurements and immersion tests in the SBF solution (37°C). The SBF solution is composed of 8.0 g/L NaCl, 0.4 g/L KCl, 0.14 g/L CaCl_2_, 0.35 g/L NaHCO_3_, 1.0 g/L C_6_H_12_O_6_ (glucose), 0.2 g/L MgSO_4_·H_2_O, 0.1 g/L KH_2_PO_4_·H_2_O, and 0.06 g/L Na_2_HPO_4_·H_2_O and pH = 7.4 [42]. The impedances of the samples were evaluated by the electrochemical impedance spectroscopy (EIS) analysis at an OCP of 10 mV from 100 kHz down to 10 MHz additionally; the potentiodynamic polarization test proceeded at a scanning rate of 5 mV/s and in the range of OCP ±1.5 V. The data curves of the tested samples for the potentiodynamic polarization and EIS experiments were analyzed through CorrView and ZSimpWin software, respectively.

### 2.5. Test of Hydrogen Evolution

All the samples were immersed in the SBF solution at 37°C for 168 hours for analyzing the corrosion behaviors of coatings on Mg alloys. The hydrogen evolution volumes of the samples in the SBF solutions were recorded every 12 hours. Three sets of samples were prepared and tested, respectively. All the tests in this paper have been repeated at least three times and five times for the data with obvious errors.

## 3. Results and Discussion

### 3.1. Phosphate Coating with the Calcium Salts Pretreatment Film

**Figure 1:**
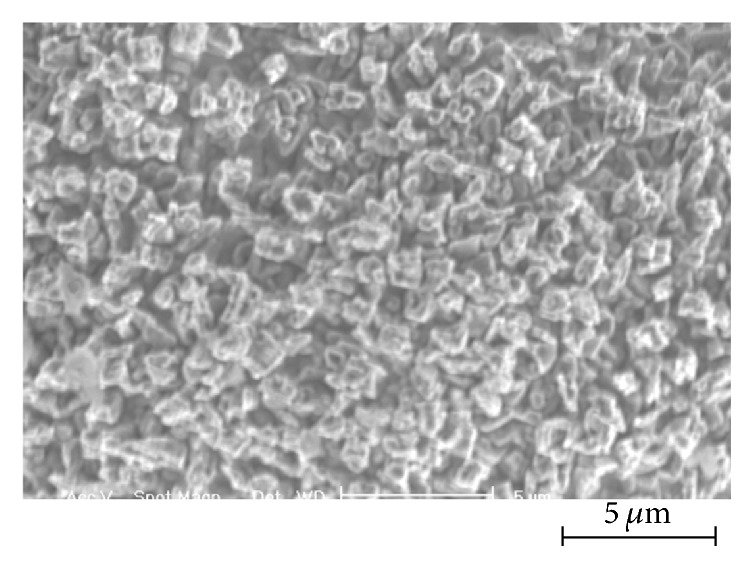
SEM of the calcium pretreatment film on the Mg alloy.

The preparation of samples was shown in Materials and Methods. Mg-5Zn-1.5Ca alloy pieces, after removing all of the oxide film, were treated by calcium salt treatment in the pretreatment solution. All of the samples were pretreated in the same solution: 1.2 mol/L CaO and 0.21 mol/L HNO_3_ at pH 1.8. SEM images of the composition of calcium salts pretreatment solution of the film are shown in Figure 1. After water rinse, samples were coated with phosphate coating in different solution; their chemical components are shown in Table 1. The manganese content in the phosphate coating was obtained by EDS. The details of the other elements of EDS are listed in Figure 3.

In Table 1, the manganese content in the phosphate coating obtained from solution containing 0.03 mol/L Mn^2+^ was less (0.96%). The manganese content in the phosphate coating obtained from solution containing 0.05 mol/L Mn^2+^ was up to 13.83%. The manganese content of phosphate coatings was increased with the Mn^2+^ addition in the phosphating solution.

Figure 1 showed SEM image of the calcium pretreatment film on the Mg-5Zn-1.5Ca alloy. After pretreatment, it can be seen that a base film with uniform and dense microstructure formed on the substrate. The pretreatment film was not sufficient to fully cover the Mg alloy substrate, but the small crystal nucleus provided a rough surface to promote the formation of the later phosphate conversion coatings and offered basic protection for the Mg alloy.

**Figure 2:**
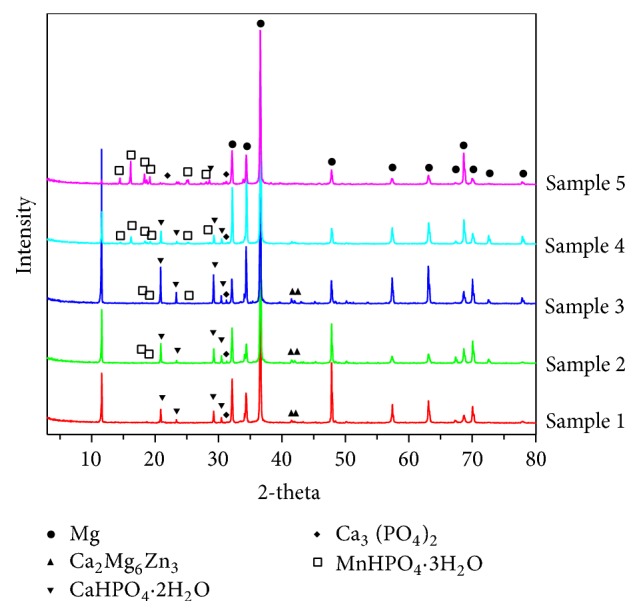
XRD analysis of phosphate coatings on samples.

Figure 2 illustrated the XRD patterns phosphate coatings with various Mn^2+^ contents on Mg-5Zn-1.5Ca alloy substrates. Dicalcium dihydrate (DCPD, CaHPO_4_·2H_2_O) and Ca_2_Mg_6_Zn_3_ were detected in CaP coating in Sample 1, and DCPD and Ca_2_Mg_6_Zn_3_ characteristic peaks remained in the phosphate coatings, but their peaks' intensity became weak in Sample 4, and they were rarely observed in Sample 5. A small quantity of tricalcium phosphate (TCP, Ca_3_ (PO_4_)_2_) that was often reported to appear in CaP coatings [27, 43] was also detected in all of the coatings. In coating of Sample 2, 0.01 mol/L Mn^2+^ was added to the phosphate coating, and manganese hydrogen phosphate trihydrate (MHPT, MnHPO_4_·3H_2_O) can be weakly detected, and its diffraction peak intensity increased with the increase of the Mn^2+^ addition in phosphate coating solution of Sample 3, Sample 4, and Sample 5.

### 3.2. Microstructure of Phosphate Coatings before Immersed Test

**Figure 3:**
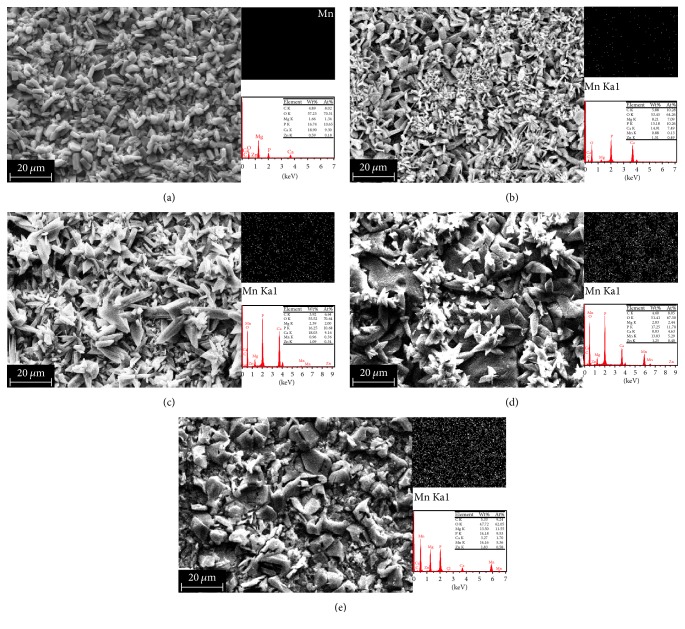
SEM surface morphology (a)–(e): phosphate coating obtained from calcium phosphating solution containing 0, 0.01, 0.03, 0.05, and 0.07 mol/L Mn^2+^ content on Samples 1–5.

The SEM surface morphology of coatings on samples was shown in Figure 3. Along with the gradual increase of Mn^2+^ content, the morphologies of the coatings show an obvious trend of variation. In all of the figures, the scattered flakes reduced on the surface morphology of samples and lumpy crystals started to appear. The flakes have been reported in researches as a typical CaP coating morphology, and the lumpy crystals could be considered a typical MnHPO_4_ structure as reported in studies of manganese phosphate coatings on Mg alloy [44]. As revealed in Figure 3(a), the surface of a CaP coating was uniform and dense, while the size of a flake approximated 10 *μ*m. From Figure 3(b), the coating of Sample 2 had smaller flakes than a CaP coating and some crystals appear. In Figures 3(c), 3(d), and 3(e), the number of crystals (MnHPO_4_·3H_2_O) increased and flakes (Ca_3_ (PO_4_)_2_ and CaHPO_4_·2H_2_O) decreased with the Mn amount increase. Especially in Figure 3(d), the flakes and crystals densely alternate with each other, so that the coating of Sample 4 can offer good protection for Mg alloy. Furthermore, most of the flakes disappeared and many crystals were cracked in Figure 3(e) for Sample 5. Obviously, the high amounts of Mn^2+^ additive make coatings containing Mn^2+^ appear to be of crystal-like structure, which probably consists of the MnHPO_4_·3H_2_O and was clearly detected by the XRD tests of Sample 4 and Sample 5, as shown in Figure 2.

### 3.3. Electrochemical Corrosion Behavior of the Samples

**Figure 4:**
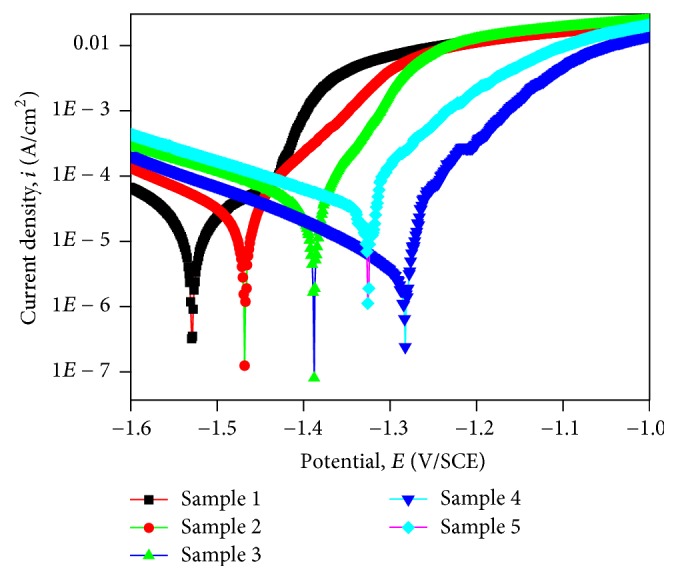
The polarization curves of coatings on Samples 1–5 in SBF solution.

Potentiodynamic polarization curves and their corresponding electrochemical parameters of the samples were shown in Figure 4 and Table 2, respectively. Figure 4 showed the corrosion potential (*E*_corr_) and corrosion current densities (*i*_corr_) of phosphate coatings. Samples with more positive *E*_corr_ would have greater potential of corrosion. As shown in Table 2, *E*_corr_ of Sample 1 and Sample 2 was more positive and *E*_corr_ of Sample 4 was the least positive and was 204 mV/SCE more negative than Sample 1. Thus, it can be deducted that, in the corrosion process, Sample 4 tended less to be corroded. *i*_corr_ was in direct proportion to corrosion rate; *i*_corr_ of Sample 4 was the lowest and was 0.036%–0.081% of other samples. As a result, Sample 4 had better anticorrosion performance and could prevent decompositions.

**Figure 5:**
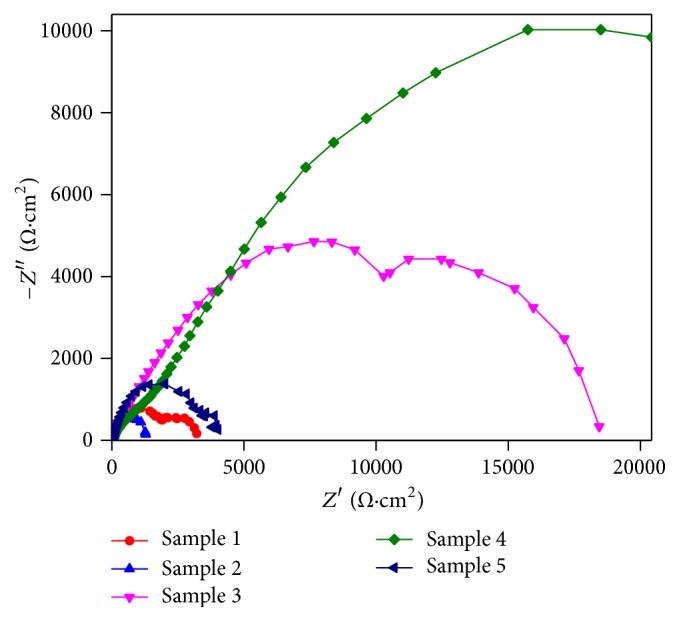
EIS patterns of coatings on Samples 1–5 in SBF solution.

**Figure 6:**
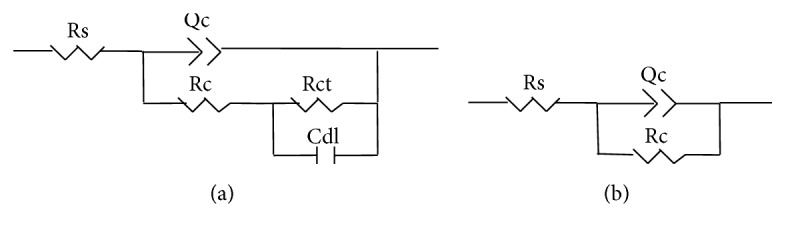
Equivalent circuits for EIS plots of (a) coatings of Samples 1, 2, and 3 and (b) coatings of Samples 4 and 5.

For further evaluation on the corrosion behaviors of the coatings, the EIS test was employed.  Figure 5 and Table 3 show the Nyquist plots and the main fitting results for the coated samples, respectively. The equivalent circuits and detailed description for the Nyquist plot in this study were similar to those in our prior research [45]. The Nyquist plot for coatings of Samples 1, 2, and 3 contained one high frequency (HF) capacitive loop and one medium frequency (MF) capacitive loop. This diagram was analyzed using the equivalent circuit in Figure 6(a), which reveals the presence of different intersurface areas with different characteristics. The HF capacitive loop was related to the dielectric properties of the coating (characterized by *R*_*c*_ and *Q*_*c*_), and *R*_ct_ was the charge transfer resistance. The second equivalent circuit shown in Figure 6(b) was used to describe the Nyquist plots for coatings of Sample 4 and Sample 5. The plots only contained one capacitance loop, which refers to the simple process at the solution/coating interface and implies that the coatings have full coverage and could supply good corrosion protection for the substrate Mg alloy. *C*_dl_ represents the electric double layer capacity at the solution/substrate interface at pinholes [1]. As compared to the base solution coated samples, the solution containing Mn^2+^ coated samples had smaller *C*_dl_ values, especially for the Sample 4 coated one. This implies that the coating of Sample 4 has denser surface structures than other coatings. The total surface resistance (*R*_total_) of the coating of Sample 4 was 36273 Ωcm^2^, which was 11.5 times *R*_total_ of the coating of Sample 1. Thus, either the potentiodynamic polarization or the EIS test indicates that the coating of Sample 4 on a Mg-5Zn-1.5Ca alloy possesses better anticorrosion ability than other coatings in this study.

### 3.4. Corrosion Behaviors of the Samples in Immersion Tests

**Figure 7:**
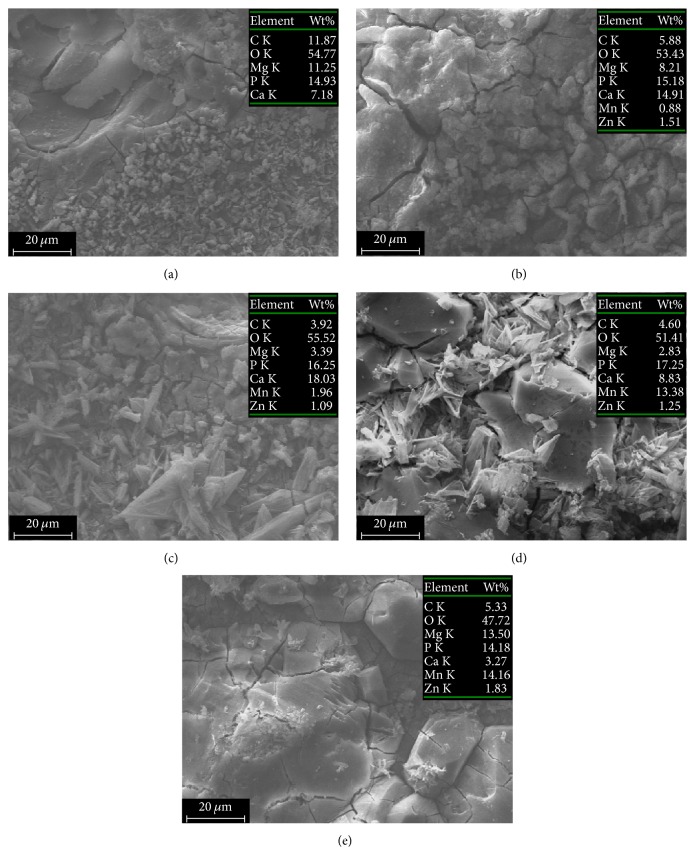
EDS and SEM of samples after being immersed in SBF for 168 hours: phosphate coating obtained from solution containing (a) 0 mol/L Mn^2+^, 0.01 mol/L Mn^2+^, 0.03 mol/L Mn^2+^, 0.05 mol/L Mn^2+^, and 0.07 mol/L Mn^2+^.

The SEM photos for the surface morphologies of the samples after being corroded by the SBF solution for 168 hours are shown in Figure 7. After being immersed in the SBF solution, the flake-like coating of Sample 1 and Sample 2 was undistinguishable, cracked, and covered by corrosion products as illustrated in Figures 7(a) and 7(b). During the immersion process, the second phase particles Ca2Mg6Zn3 acted as cathodes and caused the dissolution of the coating and the exposure of the Mg alloy substrate. On the surface of Sample 3 in Figure 7(c), the flakes were still observable and they cover most of the cracks so as to provide a better protection for the Mg alloy. The coating of Sample 4 as shown in Figure 7(d) is intact and almost free of pitting corrosion. The three components CaHPO4·2H2O, Ca3 (PO4)2, and MnHPO4·3H2O were homogenized in the coating, which may cause the change of corrosion mechanism from local corrosion to uniform corrosion. This indicates that the lumpy-crystal manganese compound offered a most improved corrosion protection for the Mg alloy. However, a small amount of corrosion cracking occurs in Figure 7(e) with the further increasing Mn content. The content of MnHPO4·3H2O in the XRD analysis in Figure 2 showed that the concentration of MnHPO4·3H2O was the highest. This is possibly caused by the stress corrosion of the lumpy coating in the simulated body fluid. Therefore, these phenomena indicate that the coating of Sample 4 provided a complemented structure that would protect Mg alloy durably.

Analysis from Figure 7 and the XRD results in Figure 2 can be made as follows:

1. The second phase particles Ca_2_Mg_6_Zn_3_ containing Ca and Zn are used as cathodes in the film layer which caused the anode of the film to dissolve and the Mg alloy matrix was exposed. Therefore, the corrosion is more severe (11.25 wt% Mg exposed) in Figure 7(a).
2. Corrosion cracking also is shown in Figure 7(b) and Figure 7(c). This was because CaHPO_4_·2H_2_O in the film contained a large amount of active element Ca. This caused dissolution corrosion, which can be seen from the calcium contents of 14.91 and 18.03 wt% in map scanning results of Figures 7(b) and 7(c), respectively.
3. The film layer in Figure 7(d) was intact and free of corrosion. CaHPO_4_·2H_2_O, Ca_3_ (PO_4_)_2_, and MnHPO_4_·3H_2_O homogenized the film, the corrosion current was evenly dispersed, and the film was more resistant to corrosion. The relative potential of these three components and the Mg alloy matrix changed, which is the cause of the change of local corrosion mechanism.
4. A small amount of corrosion cracking is shown in Figure 7(e). The content of MnHPO_4_·3H_2_O in the XRD analysis in Figure 2 showed that the concentration of MnHPO_4_·3H_2_O was higher than that of the other four samples. Maybe when concentration of the higher hard MnHPO_4_·3H_2_O in the coating surface is more than a certain value, stress corrosion crack is generated on the coating in the simulated body fluid. The mechanism would be further studied in our next work.

**Figure 8:**
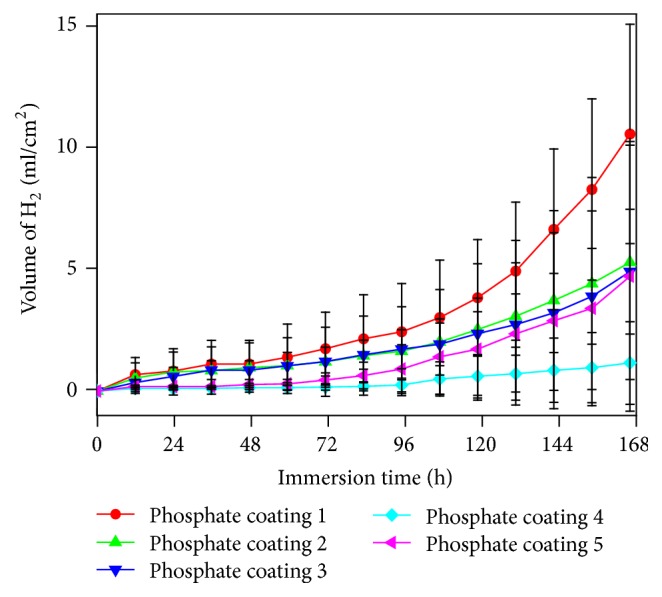
Hydrogen evolution volumes of coatings on Samples 1–5 in SBF solution for 168 hours.

The samples were, respectively, immersed in the SBF solution for 168 hours, while the hydrogen (H_2_) evolution volumes variation was shown Figure 8. Results in Figure 8 indicate that the H_2_ evolution volume of all the coatings containing Mn^2+^ was relatively lower than that of the coating of Sample 1 in the SBF, which implies better anticorrosion reaction. Coating of Sample 4 had the lowest H_2_ evolution volume in this test, which remained almost 0 ml for the first 96 hours and increased very slowly to 1.4 ml/cm^2^ measured at the 168th hour. Standard deviation in Figure 8 also demonstrates that H_2_ evolution volume of Sample 4 was more stable than that of other coatings. Hence, these results stated that the coating of Sample 4 had the promising property of lower H_2_ evolution volume applied on Mg alloy surface when served as body implant materials.

## 4. Conclusions

1. Manganese-calcium phosphate coatings were prepared on an Mg-5Zn-1.5Ca alloy in calcium phosphating solutions with different addition of Mn2+. A calcium salt pretreatment film was applied to homogenize the subsequent manganese-calcium phosphate coating.
2. Mn content in the phosphate coatings has significant influences on the coating morphology and degradation behavior. With the increasing Mn addition, the degradation resistance of the manganese-calcium phosphate coatings was gradually improved.
3. The optimum coating prepared in solution containing 0.05 mol/L Mn2+ had a uniform and compact microstructure and was composed of MnHPO4·3H2O, CaHPO4·2H2O, and Ca3(PO4)2. The electrochemical and immersion corrosion test in simulated body fluid revealed that the optimum coating had a greatly improved surface stability and degradation resistance compared to the calcium phosphate coating without Mn addition.

## Figures and Tables

**Table 1:** Composition of solutions and Mn content in the coatings of samples.

	Composition of phosphating solution (mol/L)	Mn content in phosphating coating (EDS in Figure 3) (wt%)
Sample 1	Base solution^*∗∗*^	0
Sample 2	Base solution with 0.01 mol/L Mn^2+^	0.13%
Sample 3	Base solution with 0.03 mol/L Mn^2+^	0.96%
Sample 4	Base solution with 0.05 mol/L Mn^2+^	13.83%
Sample 5	Base solution with 0.07 mol/L Mn^2+^	14.16%

^*∗∗*^Composition of base solution: 0.26 mol/L CaNO_3_·4H_2_O, 0.17 mol/L H_3_PO_4_ aqueous solution at pH 2.7.

**Table 2:** Electrochemical parameters corresponding to the polarization curves in Figure 5.

Samples	*E*_corr_ (mV/SCE)	*I*_corr_ (A/cm^2^)
Sample 1	−1529	3.548 × 10^−5^
Sample 2	−1468	2.023 × 10^−5^
Sample 3	−1387	5.400 × 10^−5^
Sample 4	−1305	7.414 × 10^−7^
Sample 5	−1325	9.063 × 10^−5^

**Table 3:** Main fitting results of Nyquist plot exhibited in Figure 6.

Samples	Rc (Ωcm^2^)	Cdl (*µ*Fcm^−2^)	Rct (Rmt) (Ωcm^2^)	Rads (Ωcm^2^)	Lads (Hcm^−2^)	Rtotal (Ωcm^2^)
Sample 1	2223	118	928	-	-	3151
Sample 2	448	13	3572	-	-	4020
Sample 3	1400	4	17030	-	-	18430
Sample 4	1843	1.3	34430	-	-	36273
Sample 5	287	5	1138	-	-	1425
